# Immune regulation through tryptophan metabolism

**DOI:** 10.1038/s12276-023-01028-7

**Published:** 2023-07-03

**Authors:** Su-Kil Seo, Byungsuk Kwon

**Affiliations:** 1 Department of Microbiology and Immunology, College of Medicine Inje University, Busan, 47392 Republic of Korea; 2 Parenchyma Biotech, Busan, 47392 Republic of Korea; 3 School of Biological Sciences, University of Ulsan, Ulsan, 44610 Republic of Korea

**Keywords:** Immunology, Cellular immunity

## Abstract

Amino acids are fundamental units of molecular components that are essential for sustaining life; however, their metabolism is closely interconnected to the control systems of cell function. Tryptophan (Trp) is an essential amino acid catabolized by complex metabolic pathways. Several of the resulting Trp metabolites are bioactive and play central roles in physiology and pathophysiology. Additionally, various physiological functions of Trp metabolites are mutually regulated by the gut microbiota and intestine to coordinately maintain intestinal homeostasis and symbiosis under steady state conditions and during the immune response to pathogens and xenotoxins. Cancer and inflammatory diseases are associated with dysbiosis- and host-related aberrant Trp metabolism and inactivation of the aryl hydrocarbon receptor (AHR), which is a receptor of several Trp metabolites. In this review, we focus on the mechanisms through which Trp metabolism converges to AHR activation for the modulation of immune function and restoration of tissue homeostasis and how these processes can be targeted using therapeutic approaches for cancer and inflammatory and autoimmune diseases.

## Tryptophan: Breakdown products influencing health and disease

The dietary essential amino acid tryptophan is converted by cellular metabolism into breakdown products that play regulatory roles in health and disease, some involving their effects on a gene control protein called the aryl hydrocarbon receptor (AHR). Su-Kil Seo at Inje University, Busan, and Byungsuk Kwon at the University of Ulsan, in South Korea, review recent insights into the role of several catabolites (breakdown products) of tryptophan in regulating the AHR. The effects of aberrant tryptophan metabolism on the AHR can promote inflammatory diseases and cancers. In addition to the body’s own metabolism, tryptophan catabolites are produced by the body’s natural microbes. Many of the effects in health and disease involve regulation of the immune system. Drugs able to interact with the AHR protein could be potential treatments for a variety of inflammatory diseases.

## Introduction

**Fig. 1:**
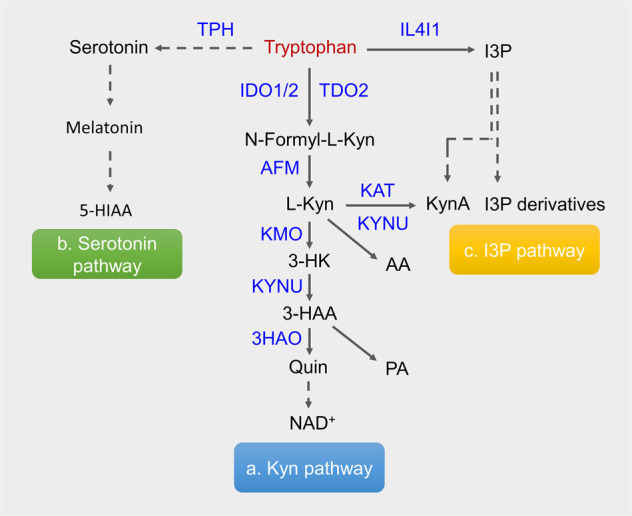
Trp catabolism in mammals. The three main catabolic pathways of Trp are shown with the enzyme metabolizing the corresponding catabolites. Dotted lines indicate that multiple steps are involved. IDO indoleamine-2,3-dioxygenase, TDO tryptophan-2,3-dioxygenase, AFM arylformamidase, L-Kyn kynurenine, KAT kynurenine amino transferase, KynA kynurenic acid, KMO kynurenine 3-monooxygenase, 3-HK 3-hydroxykynurenine, AA anthranilic acid, KYNU kynurenine, 3-HAA 3-hydroxyanthranilic acid, PA picolinic acid, 3HAO 3-hydroxyanthranilate oxidase, Quin quinolinic acid, IL4I1 interleukin-4-induced-1, I3P indole-3-pyruvate, TPH tryptophan hydroxylase, 5-HIAA 5-hydroxyindole-3-acetic acid.

The essential amino acid tryptophan (Trp) is unique in that the majority of ingested Trp is metabolized to generate bioactive compounds^1^. Trp catabolism plays an important role in the maintenance of normal physiology and can be rapidly adapted to stressful conditions. Much research has focused on the roles of Trp catabolites in barrier integrity and immunosuppression in tumors. Trp metabolites are produced by the microbiota residing in barrier organs, notably the intestines, lungs, and skin^2,3^. Endogenously produced Trp metabolites have been intensively studied because of their capacity to regulate the function of intestinal epithelial cells, where the aryl hydrocarbon receptor (AHR) is located in a central position, as dysfunction of the Trp metabolism-AHR loop is linked to inflammatory diseases and tumorigenesis^4,5^. Inflamed organs and tumors use three main pathways for Trp metabolism (Fig. 1): (1) the kynurenine (Kyn) pathway involving indoleamine 2,3-dioxygenase 1 (IDO1), IDO2 and tryptophan 2,3-dioxygenase 2 (TDO2); (2) the serotonin (5-hydroxytryptamine: 5-HT) production pathway involving tryptophan hydroxylase 1 (TPH); and (3) the indole-3-pyruvate (I3P) production pathway and its derivatives involving IL4I1^6^. The unrestrained expression of IDO1 under inflammatory conditions indicates that the kynurenine pathway is a basic negative feedback mechanism that resolves inflammation, as well as a compensatory mechanism that manages increased energy demand^7^. Trp catabolites can signal through cellular receptors, which exhibit expression that is tissue-specific and is regulated temporarily after stimulation by inflammatory cues. The receptors of Trp catabolites include AHR, α7 nicotinic acetylcholine receptor (a7nAChR), multiple ionotropic glutamate receptors, and orphan G protein-coupled receptor 35 (GPR35). AHR has binding activity to a greater number of Trp catabolites and other metabolites. Accordingly, the IDO1-Kyn-AHR axis plays a broad range of roles in the immunoregulation of inflammatory diseases and tumors. In this review article, we discuss the most recent insights regarding the role of AHR-activating Trp catabolites and their clinically translatable implications.

### The Kyn pathway

Trp metabolism occurs actively in a variety of tumors and during inflammation, as reflected by high expression levels of Trp-catabolizing enzymes (TCEs). Although nonenzymatic functions of IDO1 are implicated in the regulation of the immune response, most studies have focused on a large array of Trp metabolites that drive crosstalk between tumor, parenchymal, and immune cells or between immune cells. The Kyn pathway accounts for ~95% of Trp degradation. Two branches of research have been directed toward understanding the role of the Kyn pathway in tumor growth and inflammatory processes: (1) how Trp deprivation occurring as a result of tumor or inflammatory microenvironment formation is linked to immunosuppression and (2) how bioactive metabolites of the Kyn pathway mediate immunosuppression in an autocrine or paracrine manner. Tumors are believed to perform hijacking to leverage the immune control mechanisms by which AHR-activating Kyn and its downstream metabolites have evolved to maintain barrier functions and resolve inflammation.

IDO1 and TDO2 are constitutively expressed in various types of tumors, but their expression is inducible in immune and epithelial cells in response to inflammatory signals. This is why tumor cells are superior in depleting Trp and providing Kyn to immune cells and other stromal cells within the tumor mass. In fact, Kyn-AHR signaling involves multiple steps in tumorigenesis and metastasis^8,9^. In local and systemic inflammation, environmental and endogenous cues induce the initial expression of IDO1 in epithelial, endothelial, and myeloid cells. Later, Kyn amplifies the IDO1-Kyn-AHR loop, which leads to immunosuppression since this loop suppresses the expression of inflammatory mediators and increases the expression of anti-inflammatory mediators. There is growing interest in activating the Kyn-AHR axis to develop clinical therapeutics for inflammatory diseases. Opposite approaches have been adopted for cancer drug development. Below, we focus on how Kyn and its derivatives act on immune cells to exert their immunosuppressive effects through AHR. We also discuss the AHR-independent action of Kyn, as well as how depletion of Trp results in immunosuppression.

#### The IDO1-AHR axis in immunosuppression mediated by dendritic cells

**Fig. 2:**
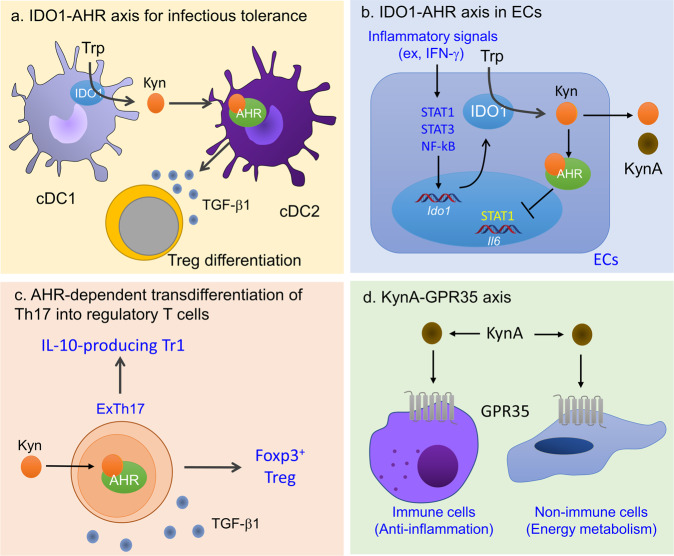
The role of the Kyn pathway in immune regulation. **a** The IDO1-AHR axis in the induction of infectious tolerance. **b** Suppression of IL6 expression by the IDO1-AHR axis in lung epithelial cells (ECs). **c** Cooperation of TGF-β1 and AHR in the transdifferentiation of Th17 cells into IL-10-producing Tr1 and Foxp3^+^ Treg cells. **d** The KynA-GPR35 axis in anti-inflammation and energy metabolism.

Dendritic cells (DCs) are responsible for initiating immunity, maintaining self-tolerance, and controlling overactivated inflammatory conditions. The induction of IDO1 and AHR expression is required for the latter function of DCs. IDO1 expression in DCs is induced by inflammatory stimuli, including type I and type II interferons, lipopolysaccharide (LPS), and extracellular and intracellular DNA^10^. This suggests that IDO1-mediated Trp catabolism serves as negative feedback regulation of inflammatory responses. The IDO1-Kyn-AHR axis adopts multiple strategies for the spread of “infectious tolerance” (Fig. 2a). First, the expression of IDO1 and AHR can be enhanced by AHR itself, thus forming an IDO1-AHR self-amplification loop to efficiently suppress immune responses. Second, Kyn released initially by IDO1-expressing type 1 conventional DCs (cDC1s) can recruit AHR-expressing cDC2s, and these cells express IDO1 through the activation of AHR^11^. Finally, the archetypical immunoregulatory cytokine TGF-β increases IDO1 expression in DCs in an arginase 1 (Arg1)-dependent manner, and the TGF-β-IDO1-AHR axis is critical for long-term self-tolerance or LPS tolerance^12–14^. This TGF-β-mediated self-amplification and maintenance of a stably tolerogenic phenotype of DCs requires phosphorylation of the immunoreceptor tyrosine-based inhibitory motif (ITIM) of IDO1 to function as a signaling molecule^12,13^. Surface counterreceptors such as CD80/CD86 and GITR ligand (TNFSF18) also induce IDO1 expression in cDCs and the production of plasmacytoid DCs (pDCs) through the production of IFN-γ and IFN-α, respectively^14–17^. Consequently, the question of how the IDO1-AHR axis drives global genetic reprogramming of tolerogenic DCs remains. The activities of AHR as a cotranscription and corepressor factor, as well as a signaling molecule, appear to determine tolerogenic DC fate. AHR activation signature genes include those relevant to inflammation and anti-inflammatory processes, suggesting that AHR induces the expression of immunoregulatory genes directly or indirectly in DCs^18^. In contrast, AHR is likely to repress the expression of inflammatory genes by inhibiting transcription factors that are critical in inflammation (for example, STAT1, AP1, and NF-κB) based on results obtained from macrophages and epithelial cells^19–21^. Studies by Gargaro et al. suggest that Kyn production by IDO1^+^ cDC1s is indispensable for self-tolerance, which is consistent with results showing that cDC1s are required for immunological tolerance induced by apoptotic cells^22,23^. cDC2s do not acquire immunoregulatory features unless tolerogenic cDC1s provide them with Kyn levels that are sufficient to induce IDO1 expression, as IL-6 induces the degradation of IDO1 protein through SOCS3 in an autocrine manner^11^. Ultimately, cDC2s appear to control cDC1s, producing large amounts of Kyn and immunosuppressive cytokines. One reason for this two-step process involved in the induction of immunological tolerance may be the numerical advantages of cDC2s. However, whether Trp catabolites prefer a paracrine versus an autocrine mechanism to generate tolerogenic cDCs should be evaluated, although single-cell RNA sequencing (scRNA-seq) analysis of splenic cDC2 supports this paracrine mechanism^24^ (BK, unpublished data). Despite accumulating evidence that AHR is indispensable for the generation of tolerogenic DCs, the AHR core module that directs a differentiation program for such tolerogenic DCs remains to be defined. This is partly due to the paucity of cell surface markers of tolerogenic DCs and the difficulty in isolating a pure population of these cells. As the production of tolerogenic DCs is induced in vitro and in vivo using various immunomodulatory substances, these cells are likely to be different in their functions and phenotypic characteristics^25^. Recently, scRNA-seq analyses of tumors have resulted in the identification of CCR7^+^ DCs that are thought to be involved in peripheral tolerance^26,27^ (this will be discussed in detail later).

#### The IDO1-AHR axis in tumor growth

There are more tumor-associated macrophages (TAMs) than DCs within tumors, and TAMs display immunosuppressive phenotypes linked to tumor growth in the majority of tumors. AHR is a key regulator of the immunosuppressive activity of TAMs. Takenaka et al. delineated the Kyn-AHR mode of immunosuppression, in which TAMs play a central role^28^. Tumor cells elicit AHR expression and activation in macrophages through the release of IL-1β/IL-6 and Kyn, respectively. Consequently, activated AHR enables macrophages to express genes required for their recruitment to tumors and polarization toward TAMs, which are able to suppress the antitumor activity of intratumoral CD8^+^ T cells.

Foxp3^+^ regulatory CD4^+^ T (Treg) cells play a central role in the immunosuppressive network of tumors. Higher levels of IDO1 and TDO2 are associated with greater immunosuppressive activity of Treg cells in tumors^29^. Kyn-activated AHR may drive Treg cell differentiation directly by inducing the expression of Foxp3^30^ or indirectly through AHR-mediated differentiation of tolerogenic DCs^31^. As TGF-β1, which is the most potent inducer of the production of Treg cells, is an AHR target gene^18^, it is likely that Treg differentiation is mediated by IDO1/TDO2-Kyn-AHR-TGF-β1. TGF-β1 and retinoic acid, which is converted from vitamin A by AHR-induced enzymes, synergistically promote FoxP3^+^ Treg differentiation^32–34^. In IDO1-overexpressing tumors, Treg cells promote tumor growth in cooperation with M2-like TAMs^29^. Clinically, the growth of tumors enriched in Trp catabolites is blocked by AHR inhibitors alone or AHR inhibitors used in combination with anti-PD-1 antibody^29^. Consistent with this finding, a novel AHR antagonist, IK-175, has been shown to act synergistically with anti-PD1 or doxorubicin to inhibit tumor growth^35^.

AHR expression is detected in intratumoral CD8^+^ T cells. Liu et al. have demonstrated that AHR-expressing CD8^+^ T cells are susceptible to T-cell exhaustion by two mechanisms^36,37^: (1) active transport of Kyn through its transporters activates AHR, which activates the transcription of PDCD1 (PD1); and (2) IL-2-mediated expression of TPH1 through STAT5 activation leads to the catabolism of Trp to 5-hydroxytryptophan (5-HTP), which is indispensable for AHR to elicit the expression of T-cell exhaustion genes such as PD1, LAG3, ENTPD1 (CD39), and HAVCR2 (TIM-3). In contrast, AHR is expressed in tissue-resident memory CD103^+^CD8^+^ T (T_RM_) cells, which are known to have potent antitumor activity, and their accumulation in tumors is associated with a better prognosis in immune checkpoint blockade (ICB)-treated non-small cell lung cancer patients^38^. Similarly, Tc22 infiltration correlates with better outcomes in ovarian cancer patients, and the generation of Tc22 cells requires AHR activation^39^. It remains to be clarified whether these two subsets have developmental links during tumorigenesis. Unlike in CD8^+^ T cells, IL-2, IL-12, and IL-15 increase the cytotoxic and IFN-γ-producing activities of natural killer (NK) cells in an AHR-dependent manner^40^. Lymphoma growth was promoted in AHR^−/−^ mice, whereas treatment with the AHR ligand 6-formylindolo[3,2-b]carbazole (FICZ) inhibited tumor growth^40^. AHR regulates the migration of NK cells by driving the expression of the ubiquitin ligase subunit ASB2, with ubiquitination of filamin A that leads to the derepression of the migratory capacity of cells^41^. In summary, the majority of studies support the immunosuppressive and protumoral roles of the IDO1-AHR axis, but they have antitumor functions in some contexts.

#### Depletion of Trp by IDO1 in tumor growth

Trp catabolism in various types of tumors leads to the depletion of Trp in the tumor microenvironment (TME), which is linked to immunosuppression. Previous studies have demonstrated that IDO1-mediated depletion of Trp in DCs can induce CD8^+^ T-cell anergy^42^ and conversion of naïve CD4^+^ T cells into Treg cells through the amino acid starvation sensor GCN2 kinase, which is activated by uncharged tRNA^43^. Although there is a correlation between TCEs (IDO1, TDO2, and IL4I1) and worse prognosis in human tumors^18^, the causal effect of Trp depletion on the immune evasion of tumors is unknown^44^. Deletion of GCN2 in myeloid-lineage cells showed that GCN2 is critical for the polarization of immunosuppressive TAMs and myeloid-derived suppressor cells (MDSCs)^45^. In addition, GCN2 activation induced by IDO1 is required for the production of IL-10 and TGF-β by splenic macrophages and the induction of peripheral tolerance to apoptotic cells^46^. Another relevant response to Trp depletion is inhibition of the mTORC1 pathway in Treg cells^47^. There is crosstalk between the GCN2 and mTORC1 pathways: (1) GCN2 prevents mTORC2 from phosphorylating AKT, thus sequentially blocking AKT and mTORC1 activation^48^; and (2) GCN2 induces expression of Sestrin2 through ATF4, which results in sustained repression of mTORC1 by blocking its lysosomal localization^49^. Inhibition of the AKT-mTORC1 loop provoked by IDO1-mediated Trp depletion is amplified for stabilization of Treg cells by the FOXO1/3A-PTEN loop^50^. Similarly, Trp depletion by IDO1 in the immunosuppressive TME inhibits mTORC1 activation, which is linked to the prevention of monocyte precursor differentiation into inflammatory MoDCs^51^. However, the role of the integrated stress response mediated by the IDO1-GCN2-mTORC1 axis in tumor cells is still unknown^52,53^.

#### The IDO1-AHR axis in tissue inflammation and autoimmunity

There is consensus that AHR is critical for controlling tissue inflammation and autoimmunity. In this section, we focus on how the IDO1-AHR axis in epithelial cells regulates tissue inflammation or autoimmunity mediated by T cells. Epithelial cells of the mucosal layer have an evolved IDO1-AHR system enabling them to manage stress conditions frequently encountered by the exterior environment. Some microbes of the microbiota and ingested food also provide AHR ligands, which play a protective role against either intestinal infection or an immune attack directed against the microbiota or other pathogens (discussed later). In allogeneic hematopoietic stem cell transplantation (HSCT) animal models, we demonstrated that IFN-γ from donor CD4^+^ T cells and inflammatory cytokines of the host, including IL-1β and TNF-α, induce the expression of IDO1 and AHR, respectively, through NF-κB activation in alveolar epithelial cells^21,54^. This cell-intrinsic IDO1-AHR pathway represses STAT1-mediated IL6 expression and subsequently prevents CD4^+^ T cells from differentiating into pathogenic Th17 cells, which are a major effector responsible for idiopathic pneumonia syndrome (IPS) (Fig. 2b). However, IDO1 and AHR have more nuanced and complex functions in intestinal homeostasis, inflammation and immunity due to the abundance of dietary AHR ligands and microbiota-derived Trp metabolites. Maintenance of the strict balance between the supply of diet-derived AHR ligands and their clearance by P450 enzymes is required for intestinal immunity^55^. CYP1A1 is a particularly important regulator of AHR ligand supply to mucosal immune cells unless ILC3- and Th17-mediated immunity to enteric infection is impaired^56–59^. AHR is also involved in the resolution of multiple processes of intestinal inflammation. Using elaborate fate-mapping mouse models, Gagliani et al. demonstrated that TGF-β1 signaling and AHR drive the transdifferentiation of Th17 cells into IL-10-producing type 1 regulatory T (Tr1) cells during the resolution phase of intestinal inflammation (Fig. 2c)^60^. It is not known whether Tr1 transdifferentiation is mediated by AHR activated by IDO1-producing endogenous ligands and whether other cytokines, such as IL-27, are involved in Th17 transdifferentiation^61–63^. In asthmatic responses, another TGF-β family member, activin-A, induces Tr1 differentiation through transactivation of the IL10 gene promoter by the IRF4-AHR complex^64^. In contrast, AHR regulates skin inflammation involving the differentiation of Langerhans cells that are capable of inhibiting the differentiation of Th2 and Tr1 cells^65^.

Accumulating evidence supports the role of the IDO1-AHR axis in the maintenance of tolerance relevant to autoimmunity. There is a paucity of evidence showing mutations of IDO1 and AHR genes that are linked to a direct cause-and-effect relationship with autoimmune diseases; however, functional defects of IDO1 caused by various factors are observed in autoimmune diseases. For example, defective IDO1 activity has been linked to the occurrence of type 1 diabetes in mice and humans^66–69^. Low expression of IDO1 in response to IFN-γ and degradation of IDO1 have been suggested as the mechanisms underlying autoimmunity. IFN-γ is a prototype inflammatory cytokine that can induce IDO1 expression in DCs and macrophages. Thus, activation of the IFN-γ-IDO1 axis by an inducer of IDO1 expression or a positive allosteric modulator is particularly effective at blocking autoimmune diseases^70,71^, whereas unresponsiveness to IFN-γ results in the opposite effects^66^. A known second cause of IDO1 impairment is the degradation of IDO1 protein by E3 ubiquitin ligase, which is mediated by SOCS3 upregulation in the inflammatory microenvironment (in particular, by IL-6 and CD28 reverse signaling)^12,72^.

Ravishankar et al. revealed that IDO1 activation plays a central role in sustaining peripheral self-tolerance to apoptotic cells, thereby causing SLE to develop when this process becomes aberrant^73^. In an apoptotic cell-induced SLE model, macrophages located in the marginal zone (MZ) of spleen phagocytized apoptotic cells and recruited FoxP3^+^ Treg cells and CD8α^+^ DCs. The expression of IDO1, IL10, and TGFB1 is induced in MZ macrophages, which also express IDO1 after the transfer of apoptotic cells^74^. Collectively, these results suggest that signaling events sequentially interconnected between MZ macrophages and cDC1s and between cDC1s and cDC2s may amplify the immunosuppressive activity of Treg cells, the final effector of systemic tolerance^11,73,74^ (B.K., unpublished results). The IDO1-AHR axis seems to act in both autocrine and paracrine fashions^11,75^.

#### AHR-independent functions of the Kyn pathway

IDO1 functions as a nonenzymatic signaling molecule that plays a critical role in peripheral tolerance in an AHR-independent manner. Pallotta et al. first demonstrated that TGF-β-induced phosphorylation of IDO1 is a key step in conferring pDCs with a long-term immunoregulatory phenotype^12^. A similar mechanism is observed in cDCs, in which SRC kinase phosphorylates IDO1 instead of Fyn in pDCs^13,14^. In both instances, IDO1 phosphorylation activates noncanonical NF-κB, which is responsible for the expression of IDO1 and TGF-β, creating a positive feedback loop that enables DCs to maintain long-term tolerance.

Kyn released from IDO1-expressing cells can be transported into and has a protective role in IDO1^-^ cells by suppressing ferroptosis^76^. Scavenging reactive oxygen species (ROS) by the Kyn downstream metabolites 3-hydroxykynurenine (3HK) and 3-hydroxyanthralinic acid (HAA) is critical for protection against ferroptosis, and these metabolites propagate anti-ferroptotic signaling by increasing Kyn import in NRF2-dependent and AHR-independent upregulation of SLC7A11^76^. The IL4I1 product I3P is also a free-radical scavenger that suppresses ferroptosis^77^.

KynA exerts its action through the G protein-coupled receptor GPR35 (Fig. 2d). Kyn-GPR35 signaling in immune cells has anti-inflammatory outcomes^78–80^. In contrast, the serotonin metabolite 5-hydroxyindoleacetic acid (5-HIAA), released by platelets and mast cells, is required for neutrophil transendothelial migration and recruitment to sites of inflammation^81^. Recent studies have demonstrated that the KynA-GPR35 axis regulates energy metabolism in adipose tissues and skeletal muscles and tissue damage control in myocardiocytes^82–84^. In summary, IDO1 catabolites may be critical for maintaining homeostasis by preventing overactivated inflammation and providing sufficient energy to manage inflammation.

### The I3P pathway

#### The role of I3P derivatives and AHR in antitumor immunity

IL4I1 is an L-amino acid oxidase, and its known substrates are L-phenylalanine (Phe), L-tyrosine (Tyr), and L-Trp^6^. IL4I1 oxidation of Phe results in the production of phenylpyruvic acid (PP), H_2_O_2_, and NH_3_, whereas IL4IL1 can catabolize Tyr and Trp to hydroxyphenylpyruvic acid (HPP) and I3P, respectively. I3P is further metabolized to indole-3-aldehyde (I3A), indole-3-lactic acid (ILA), indole-3-acetic acid (IAA), and KynA. Among these, I3A and KynA have potent AHR-activating capacity^18^. The gut microbiota also produces I3P and other indole derivatives that function as AHR ligands^2^.

**Fig. 3:**
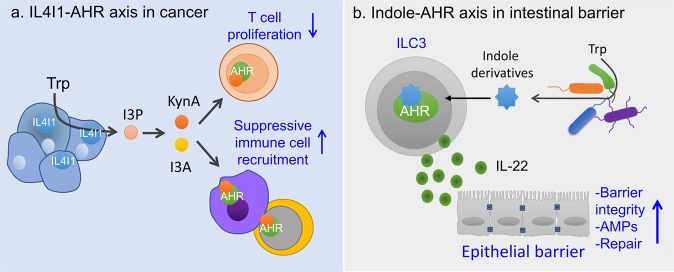
The I3P pathway in cancer and barrier function. **a** The IL4I1-AHR axis in tumor growth. **b** Microbiota-derived indole in intestinal barrier function through AHR.

Recently, Sadik et al. demonstrated that the expression of IL4I1 is greater in the majority of primary human tumor tissues than that of IDO1 and TDO2^18^. The observation that there was the highest incidence of IL4I1 expression among the seven Trp-catabolizing enzymes in AHR-associated modules in human tumors led the research team to examine whether I3P derivatives produced by IL4I1 catabolism of Trp can activate AHR. I3A and KynA have been identified as AHR agonists (Fig. 3a)^18^. It remains unclear how IL4I1 expression is regulated in tumor cells. In myeloid cells, there is accumulating evidence that IL4I1 transcription is initiated by the activation of NF-κB, STAT1, or STAT6 after stimulation with inflammatory signals^85^. Two important features of IL4I1 are worth mentioning. First, the IL4I1-AHR axis creates an amplification loop for their own expression^18^. This may explain why IL4I1 expression is persistently associated with AHR activity in several tumors. In assessments of clinical importance, although overall expression of IL4I1 and IDO1 is increased in advanced melanoma tumors after treatment with immune checkpoint blockade (ICB)^86,87^, in ICB-resistant cohorts, the levels of IL4I1 and immune checkpoint molecules, but not IDO1, are elevated^18^. Therefore, this result may explain the failure of a phase III clinical trial in advanced melanoma testing the clinical benefit of a combination of an IDO1 inhibitor and ICB^88^. Analysis of the gene ontogenies of AHR-associated modules containing IL4I1 in tumors suggests that the IL4I1-AHR axis may promote tumor growth and metastasis through tumor cell-intrinsic and paracrine mechanisms^18^. Increased levels of IL4I1 enhance tumor cell motility, a characteristic linked to tumor metastasis, in an AHR-dependent manner^18^. In addition, secreted IL4I1 or released IL4I1 metabolic products (presumably I3A and KynA) suppress activated immune cells and/or create an immunosuppressive TME (Fig. 3a)^18,89^. The mechanisms underlying IL4I1-mediated immunosuppression are diverse. The IL4I1-AHR axis plays an indispensable role in the recruitment of MDSCs to tumors and intratumoral Treg differentiation^18^. Antitumor immunity in IL4I1^−/−^ bone marrow chimeric mice indicated that IL4I1 in hematopoietic cells is sufficient to suppress the immune response to chronic lymphocytic leukemia^18^. Trp metabolite ligands of AHR released by unidentified immune cells may promote intratumoral Treg differentiation and CD8^+^ T-cell exhaustion through AHR^18,36,37^. A recent study by Maier et al. identified a subset of DCs that express high levels of IL4I1 in lung tumors^26^. These are referred to as mature DCs enriched in immunoregulatory molecules (mregDCs) and as DC3 or LAMP3^+^ DCs^90–92^. Because mregDCs display an immune-activating phenotype, mregDCs are likely to exert immunostimulatory or immunosuppressive functions in a context-dependent manner. This interpretation is supported by the results of several studies. For example, mregDCs promote the recruitment of cytotoxic CD8^+^ T cells to a distinct perivascular niche of the tumor stroma through the secretion of CXCL16, and their interaction with the recruited CD8^+^ T cells increases their cytotoxic activity through the trans-presentation of IL-15^93^. In contrast, mregDCs can induce the production of Treg cells^26^. As mregDCs are present in a wide array of tumors^92^ and normal tissues^94^, we hypothesize that they play a critical role in immune evasion in tumors and in the maintenance of peripheral immunological tolerance during immune responses. During these processes, IL4I1 metabolites may act on AHR-expressing immune cells in combination with cytokines secreted by mregDCs or other cells. For example, KynA, I3A, and IL-1B are potential inducers of Treg differentiation^26^. Our unpublished results also support the hypothesis that defects in mregDCs are associated with a decrease in Treg cell numbers and uncontrolled inflammatory responses (BK, unpublished data). In nasopharyngeal carcinoma, scRNA-seq analyses indicated that mregDCs have intimate interactions with Tregs, exhausted CD8^+^ T cells and malignant cells^95^. In the context of cancer therapy, the stratification of patients based on the expression of IDO1 and TDO2 versus IL4I1 in response to ICB is required to establish personalized strategies for therapies targeting TCEs. However, considering its critical roles in barrier biology, AHR inhibition may be carefully applied for cancer therapy, although branches of Trp catabolism converge on AHR activity in a majority of tumors.

#### Microbiota-derived Trp metabolites and AHR in barrier function and immunity

In general, metabolites of the microbiota affect multiple phases of tumor growth, treatment responses, and therapy-associated toxic effects. Recent studies have elucidated the mechanisms through which microbiota-derived indoles contribute to tumor formation. In mouse pancreatic ductal adenocarcinoma (PDAC), Hezaveh et al. demonstrated that gut *Lactobacillus* metabolism of dietary Trp to I3P derivatives can drive polarization of tumor-promoting TAMs through AHR^96^. I3P derivatives derived from the gut microbiota seemingly override their endogenous counterparts in activating AHR, thus enhancing tumor growth, suggesting the existence of a greater abundance of microbiota-derived AHR ligands in the TME^96^. However, administration of the AHR agonist indole-3-carboxaldehyde does not affect tumor growth but prevents ICB-induced intestinal damage directly or indirectly by altering gut microbiota compositions^97^.

Trp metabolites at the interface between the microbiota and host are critical for maintaining homeostasis in the body. Under inflammatory conditions, organs are exposed to microbiota-derived Trp metabolites that can activate AHR in epithelial and resident immune cells. Impaired AHR activation caused by a lack of gut bacterial metabolism of dietary Trp is considered to result in chronic tissue inflammation and autoimmunity. In the gut, AHR is expressed in multiple cells, such as intraepithelial lymphocytes (IELs), type 2 innate lymphoid cells (ILC2s), ILC3s, Th17 cells, Treg cells, and intestinal epithelial cells, and coordinates intestinal barrier functions and anti-infectious immunity (reviewed in ref. ^4^) (Fig. 3b). The repair of intestinal epithelial injury is promoted by AHR-mediated differentiation of intestinal stem cells, without which Wnt signaling is dysregulated, rendering intestinal epithelial cells vulnerable to malignant transformation^5^. In an experimental autoimmune encephalomyelitis (EAE) model, microglial cells and astrocytes are responsible for controlling brain inflammation through AHR. Trp metabolites derived from the microbiota activate AHR in astrocytes, which together with SOCS2 inhibits NF-κB transcriptional activation of inflammatory genes^98^. AHR in microglial cells promotes TGFA expression, which controls astrocytes^99^. Therefore, AHR is essential for the formation of an immunosuppressive cell network during brain inflammation. In contrast, host immune status affects the composition of the microbiota. For example, susceptibility to colitis is caused by a decreased number of *Lactobacillus* strains capable of metabolizing Trp in CARD9^−/−^ mice, which is linked to an impairment in AHR-mediated IL-22 production^100^. In a similar context, aberrant gut barrier permeability of LPS or defective production of IL-22 and secretin as a result of altered microbiota composition and Trp metabolism are associated with systemic inflammation-related metabolic syndrome, glucose dysmetabolism and hepatic steatosis^101^. Although the mechanism underlying this observation remains unknown, this study suggests that AHR agonists are targetable for the treatment of inflammatory bowel disease (IBD) and metabolic syndrome.

### The serotonin pathway

#### The serotonin pathway and AHR in antitumor immunity and inflammation

**Fig. 4:**
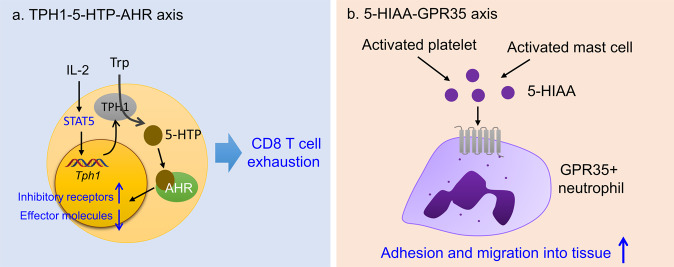
The role of the serotonin pathway in cancer and inflammation. **a** The TPH1-5-HTP-AHR axis in CD8^+^ T-cell exhaustion. **b** The 5-HIAA-GPR35 axis in neutrophil recruitment to sites of inflammation.

In the periphery, TPH1 converts Trp to 5-HTP, which is a precursor of serotonin (5-HT) and melatonin. IL-2 induces the expression of TPH1 in CD8^+^ T cells through the persistent activation of STAT5 in mouse and human tumors^37^. 5-HPH in turn activates AHR in CD8^+^ T cells, which enables them to acquire an exhaustion phenotype wherein inhibitory receptors are coordinately upregulated and cytokines and effector molecules are downregulated (Fig. 4a). This study provides insight that the TME builds up an immune evasion mechanism linked to negative feedback regulation initiated by a potent immune stimulator. Other Trp metabolites of the hydroxylation pathway, including serotonin, N-acyl serotonin, and melatonin, have anti-inflammatory and immunomodulatory properties; however, little is known about their role in antitumor immune responses^102^. However, two features of the serotonin pathway are noteworthy. First, serotonin receptors are important for angiogenesis in tumors and function as mitogenic and antiapoptotic signals, thereby promoting tumor growth^102^. Second, in human ovarian cancer, IDO inhibition elicits a metabolic adaptation that includes the conversion of Trp catabolism toward the serotonin pathway and elevated NAD^+^ production^103^. NAD^+^ suppresses T-cell proliferation and function by stimulating type A2A/A2B purinergic receptors. This result adds another layer of complexity to TCE-targeted cancer therapy.

Platelet serotonin has been known to promote the recruitment of neutrophils to sites of acute inflammation, but it does not have chemotactic capacity for neutrophils^104^. In a recent study, De Giovanni et al. demonstrated that the serotonin metabolite 5-hydroxyindoleacetic acid (5-HIAA) is responsible for neutrophil chemotaxis^81^. 5-HIAA is released by platelets and mast cells at sites of infection and binds to GPR35, which is upregulated after infection. GPR35 signaling results in increased transmigration of GPR35^+^ neutrophils through the platelet-coated endothelial layer, which are further attracted to sites of infection in response to 5-HIAA released by tissue mast cells (Fig. 4b). Serotonin regulates immune responses through its 5-hydroxytryptamine receptors expressed on various immune and nonimmune cells (reviewed in ref. ^105^).

### Therapeutic approaches targeting the Trp catabolic pathways and AHR in diseases

Theoretically, strategies to enhance AHR activity may be clinically applied for the treatment of several types of inflammatory and autoimmune diseases, whereas inhibiting AHR may be considered for cancer therapy. As mentioned previously, inflammatory signals are potent inducers of IDO1 and AHR activity; however, they are limited as therapeutics due to their general in vivo toxicities. We demonstrated that administration of an HDAC inhibitor (HDACi) potently induced IDO1 expression in the lungs of HSCT recipients in an IFN-γ-independent manner and was effective at preventing IPS^54^. HDACi-induced IDO1 expression results from the accumulation of acetyl-STAT3 after stimulation with IL-6, a cytokine responsible for the pathogenesis of IPS^106^. Thus, HDACis may be tested as prophylaxis for HSCT to prevent IPS. IFN-γ has been widely tested for priming mesenchymal stem cells (MSCs) to enhance their immunosuppressive activity; however, this priming approach has side effects. We devised a method to reduce the side effects of IFN-γ in MSCs: priming with a low dose of IFN-γ followed by bortezomib, a proteasome inhibitor, lowered the expression of class II MHC molecules, inflammatory cytokines and the cell adhesion molecule VCAM1 with intact IDO1 expression^107^. Transplantation of primed MSCs efficiently prevents acute graft-versus-host disease (GVHD) and IPS. In summary, IDO1 inducers can be targeted for the treatment of inflammatory diseases.

As AHR activation is a critical effector mechanism of immunosuppression, the development of AHR agonists may be a more promising therapeutic approach. One approach is to use natural AHR ligands. Kenison et al. developed nanoparticles containing the AHR ligand 2-(1’ H-indole-3’-carbonyl)-thiazole-4-carboxylic acid methyl ester (ITE) and epitope peptides to treat EAE and found that these nanoparticles have therapeutic effects on EAE through induction of tolerogenic DCs^108^. Recently, synthetic AHR agonists have been actively developed; however, they have inherent limitations for clinical translation because of their low efficacy, inadequate pharmacokinetics, and toxicity. Recently, AHR agonists with drug-like properties have been reported^109^. We also developed a novel synthetic indole-3-acetamide analog (PB502), a potent AHR agonist with therapeutic activity against IPS and gut GVHD^21^. PB502 effectively suppressed Th17 cells while promoting the generation of Tregs in the lungs of HSCT recipients. In an in vitro human CD4^+^ T-cell culture, PB502 exhibited synergy with TGF-β in driving the differentiation of CD4^+^ T cells toward Foxp3^+^ Treg cells. PB502 also induced marked differentiation of Treg cells under Th17 differentiation conditions. Interestingly, this drug has the ability to transdifferentiate Th17 cells into Treg cells, an important merit for IBD and psoriasis therapy (SK, unpublished result). Orally bioavailable small-molecule drugs, such as PB502, may have advantages over anti-TNF-α therapy or a range of monoclonal antibodies blocking the IL-17-IL-17R pathway. AHR agonists may notably restore the Th17/Treg balance in IBD and increase innate immunity against infection. They may have persistent therapeutic effects in patients with IBD who are resistant to anti-TNF-α therapy^110^. Finally, AHR antagonists may be considered for tumor therapy once they are guaranteed to not impair gut barrier function and immunity.

## Conclusions

This is a fruitful era for AHR research; breakthrough findings in experimental models are ready to be translated to human situations. Trp catabolites produced by the microbiota and the host are involved in a multitude of processes to maintain barrier function, control immune responses to infection and injuries, and promote tissue repair. Dysfunction of AHR or defective production of AHR ligands causes inflammatory diseases of barrier organs and even distant organs. There is crosstalk between the microbiota and the host, where the microbiota provide the host with Trp metabolites to activate AHR and AHR-mediated host programs, in turn resulting in a gut environment favorable for the survival of Trp-catabolizing microbiota. However, tumors hijack Trp catabolism to create an immunosuppressive TME for their growth, where AHR is placed at a central position. We propose that the timely administration of small-molecule AHR agonists may have therapeutic effects on inflammatory diseases, including gut/cutaneous/lung GVHD, IBD, and systemic sclerosis. DCs primed with AHR agonists and alloantigens are worth testing as prophylactic approaches to prevent GVHD and allograft rejection through the induction of tolerance. Finally, the development of a delivery system for AHR agonists and autoantigens will be urgent because successful delivery of such contents to DCs and other myeloid cells and subsequent establishment of tolerance will increase the use of therapeutic vaccines for autoimmune diseases. Deeper insights into the mechanisms of AHR action elucidated using cutting-edge technology will accelerate the applicability of AHR-targeted clinical approaches.
